# Antifungal activity of green-synthesized curcumin-coated silver nanoparticles alone and in combination with fluconazole and itraconazole against *Candida* and *Aspergillus* species

**DOI:** 10.22034/cmm.2023.345125.1456

**Published:** 2023-09

**Authors:** Seyed Mohammad Amini, Muhammad I. Getso, Shirin Farahyar, Sadegh Khodavaisy, Maryam Roudbary, Vahid Pirhajati Mahabadi, Shahram Mahmoudi

**Affiliations:** 1 Radiation Biology Research Center, Iran University of Medical Sciences, Tehran, Iran; 2 Department of Medical Microbiology and Parasitology, Faculty of Clinical Sciences, College of Health Sciences, Bayero University Kano, Kano, Nigeria; 3 Microbial Biotechnology Research Center, School of Medicine, Iran University of Medical Sciences, Tehran, Iran; 4 Department of Parasitology and Mycology, School of Medicine, Iran University of Medical Sciences, Tehran, Iran; 5 Department of Medical Parasitology and Mycology, School of Public Health, Tehran University of Medical Sciences, Tehran, Iran; 6 Zoonoses Research Center, Tehran University of Medical Sciences, Tehran, Iran; 7 Neuroscience Research Center, Iran University of Medical Science, Tehran, Iran

**Keywords:** Curcumin, Nanoparticles, Antifungal agents, *Candida*, *Aspergillus*

## Abstract

**Background and Purpose:**

Regarding the wide-spectrum antimicrobial effects of curcumin and silver, this study aimed to evaluate the antifungal activity of green-synthesized curcumin-coated silver nanoparticles (Cur-Ag NPs) against a set of *Candida* and *Aspergillus* species.

**Materials and Methods:**

Cur-Ag NPs were synthesized by mixing 200 µL of curcumin solution (40 mM) and 15 mL of deionized water. The mixture was stirred for 3-5 min, followed by the addition of 2.5 mL of silver nitrate solution (2.5 mM). The resulting solution was incubated for 3 days. Antifungal susceptibility of 30 fungal isolates of *Aspergillus* and *Candida* to fluconazole and itraconazole, as well as the activity of Cur-Ag NPs against the isolates, were determined, both alone and in combination, using broth microdilution according to the Clinical and Laboratory Standards Institute guidelines.

**Results:**

Cur-Ag NPs demonstrated promising antifungal activity, particularly against *Candida* species. The geometric mean value of the minimum inhibitory concentration of Cur-Ag NPs was significantly lower than that of fluconazole for all the studied fungi. Similarly, it was lower than those of itraconazole in *C. albicans* and *A. fumigatus*. The minimum fungicidal concentrations of Cur-Ag NPs were markedly better than those of fluconazole but still inferior to those of itraconazole.

**Conclusion:**

Cur-Ag NPs demonstrated indisputable antifungal activity and great potential that can be harnessed to combat fungal infections, particularly those caused by azole-resistant strains of *Aspergillus* and *Candida*.

## Introduction

The parallel increase in the prevalence of fungal infections and antifungal resistance leads to prolonged hospitalization and increased healthcare costs, overall morbidity, and mortality associated with these diseases [ 1 , 2 ]. Emergence of resistant strains and the side effects of currently available antifungal drugs necessitate the search for new targets and improved therapeutic agents [ 3 ]. Given the limited number of available antifungal drugs, researchers are dedicated to developing new, nontoxic, broad-spectrum, and cost-effective antifungal agents derived from naturally occurring materials [ 4 , 5 ]. 

Curcumin is a bioactive molecule derived from naturally occurring tropical plants, specifically *Curcuma longa*, which belongs to the *Zingiberaceae* family. Traditionally, curcumin has been used to treat inflammatory diseases and bacterial infections [ 6 ]. Recently, researchers have conducted several investigations on curcumin due to its potential health benefits, including its antioxidant, anti-inflammatory, anti-infective, and anticancer effects [ 7 , 8 ]. However, poor stability and limited bioavailability of curcumin after gastrointestinal administration have made it unpopular in clinical settings. To overcome these challenges, adjuvant approaches, such as nano-formulation, are being explored to harness the clinical benefits of this biomolecule. Various encapsulation techniques are being employed to enhance its nutritional and therapeutic benefits, with nano- and micro-encapsulation being particularly desirable [ 9 ]. Curcumin exhibits antifungal activity through mechanisms such as the release of reactive oxygen species, alteration of the ergosterol biosynthesis pathway, impairment of hyphal development, disruption of HSP90, and modulation of multidrug efflux pumps [ 10 , 11 ].

In addition, silver ions and silver nanoparticles (Ag NPs) exhibit a broad spectrum of antimicrobial activities with relatively low toxicity to mammalian cells. Reports confirm that exposure to Ag NPs leads to distortions and damages in microbial membrane architecture. These nanoparticles demonstrate significant antifungal effects by generating reactive oxygen species (ROS), singlet oxygen, and hydroxyl radicals (OH), which result in cellular oxidative damage [ 7 , 12 , 13 ].

While several reports have indicated the usefulness of combination therapy to overcome antifungal resistance, the fungicidal effects of conventional antifungal drugs in combination with green-synthesized curcumin-coated silver nanoparticles (Cur-Ag NPs), have not been investigated. Therefore, this study aims to assess the antifungal activity of green-synthesized Cur-Ag NPs alone and in combination with fluconazole and itraconazole. 

## Materials and Methods

### Fungal isolates

In this study, a total of 30 fungal isolates were included, comprising *Aspergillus fumigatus* (n=6), *Aspergillus flavus* (n=6), *Candida albicans* (n=6), *Candida parapsilosis* (n=6), and *Candida krusei* (n=6). These isolates were previously identified using PCR-sequencing of the β-tubulin region (for *Aspergillus* spp.) or the ITS1-5.8S-ITS2 region (for *Candida* spp.).

### Green synthesis and characterization of curcumin-coated silver nanoparticles (Cur-Ag NPs)

Curcumin (C21H20O6, 65%, Sigma-Aldrich, USA) and silver nitrate (AgNO3 99.9%, Dr. Mojallali chemical company, Iran) were purchased, and stock solutions were prepared at concentrations of 40 mM and 2.5 mM, respectively. To synthesize Cur-Ag NPs, 200 µL of the curcumin stock solution was added to 15 mL of deionized water (Barnstead E-PureTM18.3 MX water), and the pH was adjusted to 10. The solution was stirred for 3-5 minutes, after which 2.5 mL of the silver nitrate stock solution was added. The mixture was left for three hours, followed by three days of incubation to complete the reaction. The synthesized nanoparticles were then washed multiple times to remove any remaining unreacted curcumin or silver ions. This washing step involved a series of centrifugation steps and replacement of the supernatant with deionized water. The stability, morphology, size distribution, and inductively of the Cur-Ag NPs in physiological solutions were determined using a SPEKOL 2000 double-beam UV-visible spectrophotometer (Analytik Jena, UK) and transmission electron microscopy (TEM, ZeissEM10C-100 KV Germany electron microscope). The concentration of the final solution of Cur-Ag NPs was analyzed using inductively coupled plasma-atomic emission spectroscopy (ICP-OES) (Vista-Pro, Varian, Palo Alto, USA) [ 14 ].

### Antifungal susceptibility testing

The susceptibility profile of the fungal isolates to fluconazole and itraconazole was determined following the guidelines provided by the Clinical and Laboratory Standards Institute (CLSI) M27 (4^th^ edition) and CLSI M38 3^rd^ edition for *Candida* and *Aspergillus* species, respectively [ 15 , 16 ]. In brief, stock solutions of the antifungal drugs were prepared in dimethyl sulfoxide (DMSO) and serially diluted using RPMI 1640 (Gibco, UK) to create final concentration ranges of 0.0312–16 µg/mL and 0.125–64 µg/mL for itraconazole and fluconazole, respectively. From the various concentrations of each antifungal drug, 100 µL was dispensed into columns 1 to 10 of 96-well microplates in a descending concentration manner.

Fungal suspensions were prepared from fresh colonies and adjusted to the recommended density using the spectrophotometric method as outlined by the Clinical and Laboratory Standards Institute guidelines. Subsequently, 100 µL of the suspension was added to all wells of the microplates, except for the negative control wells (fungi-free). The plates were then incubated at 35 °C, and the results were visually observed after 24-48 h. The minimum inhibitory concentration (MIC) was defined as the lowest concentration of drugs that resulted in a prominent decrease (approximately 50%) in growth, except for itraconazole against *Aspergillus* species, where complete (100%) inhibition was utilized. The MIC values were interpreted as susceptible/resistant based on the established clinical breakpoints. Otherwise, isolates were categorized as wild type/non-wild type according to their epidemio-logical cutoff values. Moreover, *Candida parapsilosis* ATCC 22019 and *Candida krusei* ATCC 6258 strains were used for quality control. It should be mentioned that all experiments were conducted in duplicate.

The antifungal activity of synthesized curcumin-coated silver nanoparticles (Cur-Ag NPs) against *Aspergillus* and *Candida* isolates was assessed using the same method described above. The final concentrations of the nanoparticles ranged from 0.5 µg/mL to 256 µg/mL. The MICs were determined as the lowest concentrations that led to a significant decrease (approximately 50%) in growth.

### Minimum fungicidal concentration of antifungal drugs and Cur-Ag NPs

To determine the minimum fungicidal concentrations (MFC) of fluconazole, itraconazole, and Cur-Ag NPs, 10 µL from sub-MIC concentrations were cultured on Sabouraud dextrose agar plates (Ibersco, Iran). The lowest concentrations that resulted in complete inhibition of growth (no colonies) were considered MFC.

### In vitro combination of Cur-Ag NPs with antifungal drugs

In this step, one isolate from each species of the genus *Candida* with the highest MIC of fluconazole and one isolate from each species of the genus *Aspergillus* with the highest MIC of itraconazole was selected. The antifungal activity of Cur-Ag NPs when combined with itraconazole (against *Aspergillus* species) or fluconazole (against *Candida* species) was determined using a checkerboard method [ 17 ]. Range of concentrations used in this step was selected based on the MICs of nanoparticles and antifungal drugs when tested alone. Test plates were prepared as described previously [ 18 - 20 ]. In order to interpret the results, the fractional inhibitory concentration index (FICI) was calculated based on the following formula:

FICI = (MIC drug A in combination/MIC drug A alone) + (MIC drug B in combination/MIC drug B alone)

 The interaction was considered synergistic (FICI<0.5), indifferent (FICI≥0.5–4), or antagonistic (FICI>4) [ 17 ]. 

## Results

### Characteristics of Silver nanoparticles

**Figure 1:**
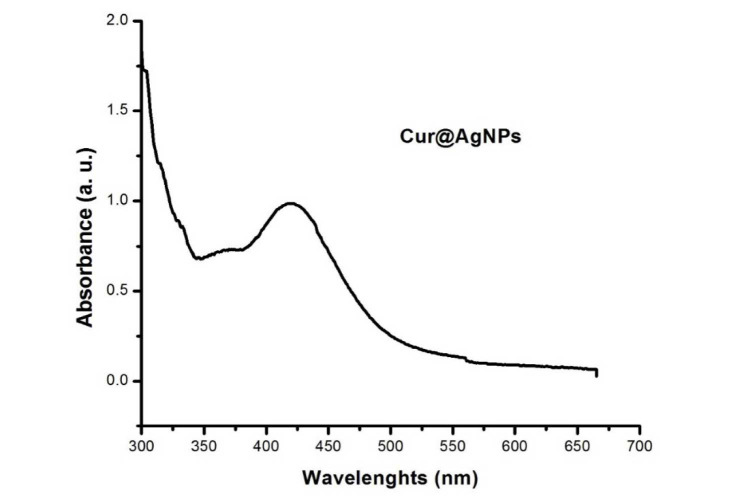
Result of ultraviolet-visible spectroscopy of curcumin-coated silver nanoparticles showing a peak at 420 nm wavelength

**Figure 2:**
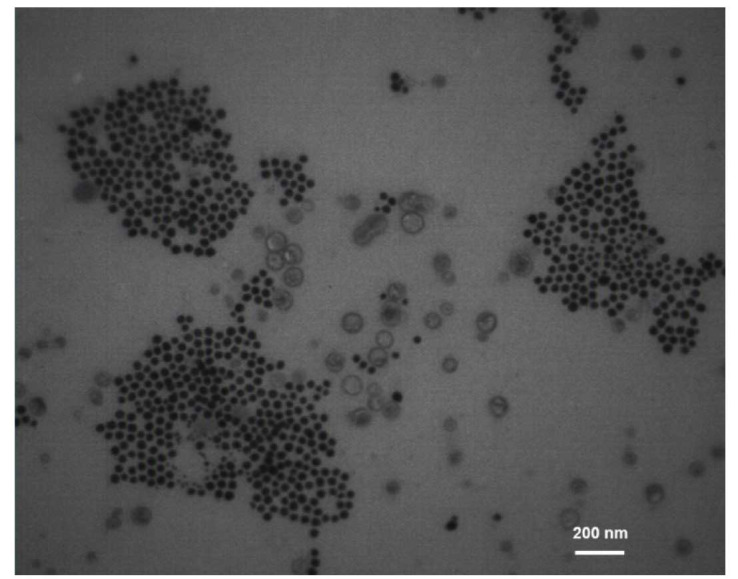
Transmission electron microscopy micrograph of curcumin-coated silver nanoparticles showing their spherical morphology

Successful synthesis of Cur-Ag NPs was confirmed by the results of ultraviolet-visible spectroscopy, where the expected surface plasmon resonance peak was detected at a wavelength of 420 nm (Figure 1). Additionally, transmission electron microscopy (TEM) micrograph analysis revealed that the morphology of the nanoparticles was spherical, with a mean ± SD value of 29.1 ± 5.6 nm (Figure 2).

### Antifungal activity of fluconazole, itraconazole, and Cur-Ag NPs

Results of antifungal susceptibility testing showed that the majority of yeasts (n=15, 83%) were fluconazole-resistant. Resistance to itraconazole was also noted in 4 (22%) *Candida* and 7 (58%) *Aspergillus* strains (Table 1). Cur-Ag NPs demonstrated remarkable antifungal activity, especially against *Candida* species. The geometric mean (GM) MIC of Cur-Ag NPs was much lower than that of fluconazole against all the studied fungi. These values were also lower than those of itraconazole in some instances, i.e. against *C. albicans* and *A. fumigatus*. Results are shown in Table 1.

**Table 1:** Results of antifungal susceptibility testing for fluconazole, itraconazole, and curcumin-coated silver nanoparticles against Candida and Aspergillus species

Fungal species (number of isolates)	Antifungal drugs	MIC range (µg/mL)	GM	Distribution of MICs (µg/mL)*													N of R/NWT
				0.06	0.125	0.25	0.5	1	2	4	8	16	>16	32	64	>64	
*Candida albicans* (6)	Fluconazole	≥ 64	>64			1									1	4	5
	Itraconazole	0.06–>16	1.97	2			1						3				3
	Cur-Ag NPs	0.5–8	1.41				2	1	2		1						-
*Candida krusei* (6)	Fluconazole	≥ 64	>64												4	2	6
	Itraconazole	0.25–16	0.5			5						1					1
	Cur-Ag NPs	0.25–1	0.63			1	2	3									-
*Candida parapsilosis* (6)	Fluconazole	0.5–16	6.35				1		1			4					4
	Itraconazole	0.06–0.25	0.12	2	2	2											0
	Cur-Ag NPs	0.125–1	0.56		1		2	3									-
*Aspergillus fumigatus* (6)	Fluconazole	>64	>64													6	6
	Itraconazole	0.5–16	4				1		2		1	2					5
	Cur-Ag NPs	1–4	2.52					1	2	3							-
*Aspergillus flavus* (6)	Fluconazole	>64	>64													6	6
	Itraconazole	0.5–2	1				2	2	2								2
	Cur-Ag NPs	4–8	4.49							5	1						-

MIC: minimum inhibitory concentration, GM: geometric mean, R: resistant, NWT: non-wild type, Cur-Ag NPs: curcumin-coated silver nanoparticles

* >16 and >64 are for off-scale results of itraconazole and fluconazole, respectively. These MICs were changed to the next higher MIC in calculation of GM.

Regarding the minimum fungicidal concentrations, the results of Cur-AgNPs were much better than those of fluconazole but inferior to those of itraconazole, as summarized in Table 2. Cur-AgNPs were more effective against *Candida* species with GM MICs ranging from 11.31 to 28.51 µg/mL, compared to *Aspergillus* species with GM MICs of 71.84 µg/mL and 90.51 µg/mL against *A. flavus* and *A. fumigatus*, respectively. 

**Table 2:** Minimum fungicidal concentrations of fluconazole, itraconazole, and curcumin-coated silver nanoparticles against *Candida* and *Aspergillus* species

Fungal species (number of isolates)	Antifungal drugs	MFC range (µg/mL)	GM	Distribution of MICs (µg/mL)										
				1	2	4	8	16	>16	32	64	>64	128	256
*Candida albicans* (6)	Fluconazole	1–>64	57.01	1								5		
	Itraconazole	1–>16	8.98	1	1		1		3					
	Cur-Ag NPs	16–32	28.51					1		5				
*Candida krusei* (6)	Fluconazole	>64	>64									6		
	Itraconazole	2–>16	4.49		2	3			1					
	Cur-Ag NPs	2–32	11.31		1		1	3		1				
*Candida parapsilosis* (6)	Fluconazole	2–>64	40.32		1		1					4		
	Itraconazole	1–4	2.52	1	2	3								
	Cur-Ag NPs	8–128	22.63				3			1	1		1	
*Aspergillus fumigatus* (6)	Fluconazole	>64	>64									6		
	Itraconazole	4–>16	14.25			1	2		3					
	Cur-Ag NPs	32–128	90.51							1	1		4	
*Aspergillus flavus* (6)	Fluconazole	>64	>64									6		
	Itraconazole	2–16	5.04		1	3	1	1						
	Cur-Ag NPs	16–256	71.84					1		1	1		2	1

MFC: minimum fungicidal concentration, MIC: minimum inhibitory concentration, GM: geometric mean, Cur-Ag NPs: curcumin-coated silver nanoparticles

### In vitro combination of curcumin-coated silver nanoparticles with antifungal drugs

Results of the checkerboard method revealed that curcumin-coated silver nanoparticles interact synergistically with fluconazole against *Candida* species (Table 3). Interestingly, these nanoparticles could potentiate the antifungal activity of fluconazole and reverse fluconazole resistance in all *Candida* strains, i.e. the MICs of fluconazole when combined with nanoparticles were within the susceptible range. In contrast to fluconazole, the combination of curcumin-coated silver nanoparticles with itraconazole resulted in indifferent interaction against *Aspergillus* species (Table 3). 

**Table 3:** Results of *in vitro* combination of curcumin-coated silver nanoparticles with antifungal drugs against *Candida* and *Aspergillus* species

Fungal species	Susceptibility profile	Type of combination	MICs alone	MICs in combination	FICI	Interpretation
*Candida albicans*	FLU-resistant	Cur-AgNPs /FLU	0.5/>64	<0.06/<0.125	0.06	Synergism
*Candida krusei*	FLU-resistant	Cur-AgNPs /FLU	1/64	<0.06/2	0.06	Synergism
*Candida parapsilosis*	FLU-resistant	Cur-AgNPs /FLU	0.5/16	0.06/1	0.18	Synergism
*Aspergillus fumigatus*	ITR-non-wild type	Cur-AgNPs /ITR	16/16	16/2	1.125	Indifferent
*Aspergillus flavus*	ITR-wild type	Cur-AgNPs /ITR	16/1	8/1	1.5	Indifferent

FICI: fractional inhibitory concentration index, Cur-AgNPs: curcumin-coated silver nanoparticles, FLU: fluconazole, ITR: itraconazole, MIC: minimum inhibitory concentration

## Discussion

Several factors, such as the continuous rise in fungal infections, the increasing population of patients at risk, and the soaring prevalence of antifungal resistance due to emerging drug-resistant strains, contribute to the treatment complexity and failure [ 21 ]. Usage of natural products, like curcumin, and the combination of compounds or drugs has drawn the attention of many researchers. For this purpose, the present study evaluated the antifungal activity of green-synthesized Cur-Ag NPs and its potential effects in combination with conventional antifungal drugs.

Several research projects have been previously conducted to evaluate the antifungal activity of curcumin nanoformulations, predominantly against drug-resistant fungal strains [ 22 - 24 ]. In the current study, it was found that Cur-Ag NPs have promising antifungal activities, especially against Candida species with GM MIC values ranging from 11.31 to 28.51 µg/mL and *A. flavus* and *A. fumigatus* with GM MICs of 71.84 µg/mL and 90.51 µg/mL, respectively. Similarly, Paul et al. indicated that Cur-Ag NPs exhibited excellent antifungal activity against all *Candida* species evaluated in their study, with MIC values ranging from 31.2 µg/mL to 250 µg/mL [ 22 ]. 

In the present study, the GM MICs of Cur-Ag NPs were much lower than those of fluconazole against all the studied fungi. These values were also lower than those of itraconazole against *C. albicans* and *A. fumigatus*. Similarly, a previous study demonstrated that curcumin showed better antifungal activity against clinical isolates of *Paracoccidiodes braziliences* and *Sporothrix schenckii*, compared to commercial fluconazole. In addition, the authors found that curcumin was more potent than fluconazole to inhibit the adhesion of the studied *Candida* species to buccal epithelial cells of HIV/AIDS patients [ 23 ]. Promising inhibitory activity of Cur-Ag NPs is not limited to fungal agents, but rather seems to have a wide spectrum. This property was highlighted in different studies. For example, Maghimaa et al. evaluated the antimicrobial activity and wound healing potential of green-synthesized Cur-Ag NPs loaded in cotton fabric, and the results showed a remarkable decrease in the growth rate of pathogenic microbes, especially *Staphylococcus aureus*, *Pseudomonas aeruginosa*, *Streptococcus pyogenes*, and *C. albicans* [ 25 ]. 

Badirzadeh et al. showed the therapeutic effect and the safety of Cur-Ag NPs in the treatment of cutaneous leishmaniasis *in vitro* and in an animal model. They found Cur-Ag NPs to exhibit good anti-leishmania activity at concentrations much lower than the toxic level, and they were safely tolerated by the animal model at therapeutic concentrations [ 14 ]. 

Moreover, potent anti-infective properties of Cur-Ag NPs against gram-negative and gram-positive bacteria [ 12 , 26 ], viruses [ 27 ], and many other agents [ 28 - 31 ], and limited cellular toxicity in hosts are being reported. Antifungal effects of nanocurcumin are observed even with the topical formulation. Anwar et al. (2023) compared the efficacy of topical nanocurcumin and conventional nystatin in the treatment of oral candidiasis and found that nanocurcumin has a good antifungal effect as nystatin; however, its therapeutic efficacy requires a longer time to appear, compared to nystatin [ 32 ]. 

Combined use of antifungal drugs or the combination of antifungal drugs with natural products is promising, even against multidrug-resistant fungi [ 33 ]. In the current study, Cur-Ag NPs interacted synergistically with fluconazole against Candida species. More noteworthy, these nanoparticles could potentiate the antifungal activity of fluconazole and reverse fluconazole resistance in all the studied *Candida* strains. In contrast, itraconazole combined with Cur-Ag NPs resulted in indifferent action against *Aspergillus* species. Sharma et al. demonstrated that curcumin interacts synergistically with fluconazole and amphotericin B against *Candida* species via the generation of reactive oxygen species [ 34 ]. 

Theoretically, therapeutic synergism between nontoxic natural products, such as curcumin and conventional antifungals could be an alternative approach to the treatment of fungal infections. Further investigation is required to prove this. 

Although the present study provided valuable data regarding the antifungal activity of Cur-Ag NPs, it was performed on a limited number of isolates. Moreover, the mechanistic background behind the antifungal activity (inhibitory or fungistatic) or the synergistic interactions was not investigated. These limitations guarantee the need for future studies on Cur-Ag NPs.

## Conclusion

According to the promising activity of Cur-Ag NPs in this study, these nanoparticles might reduce the gaps created by the use of conventional antifungal drugs, especially the undesirable side effects and the emergence of resistance. Altogether, Cur-Ag NPs showed indisputable antifungal potential and a great prospect in combating the rising problem of emerging antifungal resistance and healthcare costs. However, these findings need further evaluation in animal and human settings.
